# Orthosteric- versus allosteric-dependent activation of the GABA_A_ receptor requires numerically distinct subunit level rearrangements

**DOI:** 10.1038/s41598-017-08031-9

**Published:** 2017-08-10

**Authors:** Jahanshah Amin, Meena S. Subbarayan

**Affiliations:** Department of Molecular Pharmacology and Physiology, Morsani College of Medicine, University of South Florida, Tampa, Florida 33612 USA

## Abstract

Anaesthetic molecules act on synaptic transmission via the allosteric modulation of ligand-gated chloride channels, such as hetero-oligomeric α_1_β_2_γ_2_ GABA_A_ receptors. To elucidate the overall activation paradigm via allosteric versus orthosteric sites, we used highly homologous, but homo-oligomeric, ρ_1_ receptors that are contrastingly insensitive to anaesthetics and respond partially to several full GABA α_1_β_2_γ_2_ receptor agonists. Here, we coexpressed varying ratios of RNAs encoding the wild-type and the mutated ρ_1_ subunits, which are anaesthetic-sensitive and respond with full efficacy to partial GABA agonists, to generate distinct ensembles of receptors containing five, four, three, two, one, or zero mutated subunits. Using these experiments, we then demonstrate that, in the pentamer, three anaesthetic-sensitive ρ_1_ subunits are needed to impart full efficacy to the partial GABA agonists. By contrast, five anaesthetic-sensitive subunits are required for direct activation by anaesthetics alone, and only one anaesthetic-sensitive subunit is sufficient to confer the anaesthetic-dependent potentiation to the GABA current. In conclusion, our data indicate that GABA and anaesthetics holistically activate the GABA_A_ ρ_1_ receptor through distinct subunit level rearrangements and suggest that in contrast to the global impact of GABA via orthosteric sites, the force of anaesthetics through allosteric sites may not propagate to the neighbouring subunits and, thus, may have only a local and limited effect on the ρ_1_ GABA_A_ receptor model system.

## Introduction

The excitatory and inhibitory ligand-gated ion channels play a central role in the control of synaptic transmission in the central nervous system. Extensively diversified GABA_A_ receptors (γ-aminobutyric acid-gated chloride channels) constitute a principal component of the inhibitory processes^1–4^. GABA_A_ receptors are pentamers that can exist as either hetero- or homo-oligomers. Various combinations of homologous subunits with a nomenclature of α (six isoforms), β (three isoforms), γ (three isoforms), δ, ε, π, and θ constitute the hetero-oligomeric receptor-channels (e.g., α_1_β_2_γ_2_ receptors); however, the ρ subunits (three isoforms) aggregate to assemble the homo-oligomeric ρ GABA_A_ receptors (previously known as GABA_C_ receptors, e.g., ρ_1_ receptor)^2^. In addition to the GABA-dependent activation via the orthosteric site, structurally diverse compounds, such as anaesthetics, can modulate the GABA-dependent activity of receptors and can directly activate GABA_A_ receptors allosterically, except for the ρ_1_ receptor, which is insensitive to anaesthetics^5–11^. A detailed picture has emerged regarding the positions and the amino acid side chain requirements for anaesthetic- versus GABA-dependent action. Specifically, GABA and anaesthetics act on separate sites, and the crucial amino acids that are required for the effects of GABA are located in the extracellular domain of the receptor, while the residues that are needed for the effects of anaesthetics are situated mainly in the second (TM_2_) and third (TM_3_) transmembrane domains. Asn265 in the TM_2_ and Met286 in the TM_3_ of the β_2/3_ subunit have been shown to be the crucial residues for the anaesthetic-dependent action on the hetero-oligomeric GABA_A_ receptor. Converse mutations of the corresponding residues in the ρ_1_ subunit (Ile307-TM_2_ and Trp328-TM_3_) confer sensitivity to structurally distinct classes of anaesthetics, such as barbiturates and benzodiazepines (e.g., diazepam), to the ρ_1_ receptor^7, 12–37^. The imparted TM action of diazepam on the ρ_1_ receptor occurs in the micromolar concentration range (also demonstrated in α_1_β_2_γ_2_) and is distinct from the high-affinity nanomolar effects of the benzodiazepine located at the α-γ interface in the extracellular domain of the α_1_β_2_γ_2_ receptors^8, 22, 38^. Studies on the ρ_1_ receptor have demonstrated flexibility in the amino acid side chain requirements for the crucial TM_2_ and TM_3_ anaesthetic residues to confer anaesthetic sensitivity. By contrast, even conservative mutations in the crucial amino acids (e.g., Tyr to Phe) in the GABA-dependent activation domain markedly impair the GABA sensitivity^13, 14, 39^.

The five subunits of a single GABA_A_ receptor exists as a dynamic ensemble that shift between tense and relaxed states in the absence of GABA^40–48^. GABA binds preferentially to the relaxed state in the orthosteric site of the receptor domain, leading to a systematic stabilization of the channel in the open configuration. Studies have elucidated the number of GABA-binding steps that are crucial for maintaining the channel in an open configuration, which is the mechanism underlying the GABA-dependent activation^1, 49–52^. For hetero-oligomeric GABA_A_ receptors, such as α_1_β_2_γ_2_, the number of GABA binding steps required to stabilize the channel in its open mode has been shown to be two. In comparison, the number of binding steps (with one GABA binding per subunit) required to maintain the channel in an open configuration in the homo-oligomeric ρ_1_ receptor is three^50, 51^. Despite a relatively thorough understanding of the processes involved in the GABA-dependent activation via the orthosteric sites, the mechanism by which anaesthetics act allosterically to open or modulate the GABA_A_ receptors has remained an enigma^5, 7, 11, 53–55^.

In this study, we have shown that specific mutations in the TM_2_ and TM_3_ domains of the ρ_1_ subunit not only confer marked sensitivity to several classes of diverse anaesthetics, including midazolam, diazepam, barbiturate pentobarbital, ketamine, propofol, and etomidate, but also impart the full efficacy of the known partial GABA agonists to the ρ_1_ receptor. We coexpressed complementory RNAs (cRNAs) corresponding to the wild-type and the anaesthetic-sensitive ρ_1_ subunits at different ratios to determine the number of anaesthetic-sensitive subunits that are crucial for 1) imparting the full efficacy of partial GABA agonists, 2) conferring anaesthetic sensitivity at the level of direct activation, and 3) conveying anaesthetic-dependent potentiation of the GABA currents. We then demonstrate that, in the pentamer, the number of anaesthetic-sensitive ρ_1_ subunits needed to impart full efficacy to the partial GABA agonists is three. By contrast, the number of anaesthetic-sensitive subunits needed for direct activation by anaesthetics alone is five, and the number of anaesthetic-sensitive subunits needed to confer the anaesthetic-dependent potentiation to the GABA current is one. Given that GABA-induced subunit level rearrangements to open the channel appear to be different than those that are induced by anaesthetics, the potential characteristics of the interactions between ligands and orthosteric versus allosteric sites of the GABA_A_ receptors are discussed.

## Results

### Imparting sensitivity to intravenous anaesthetics to the ρ_1_ receptor

**Figure 1:**
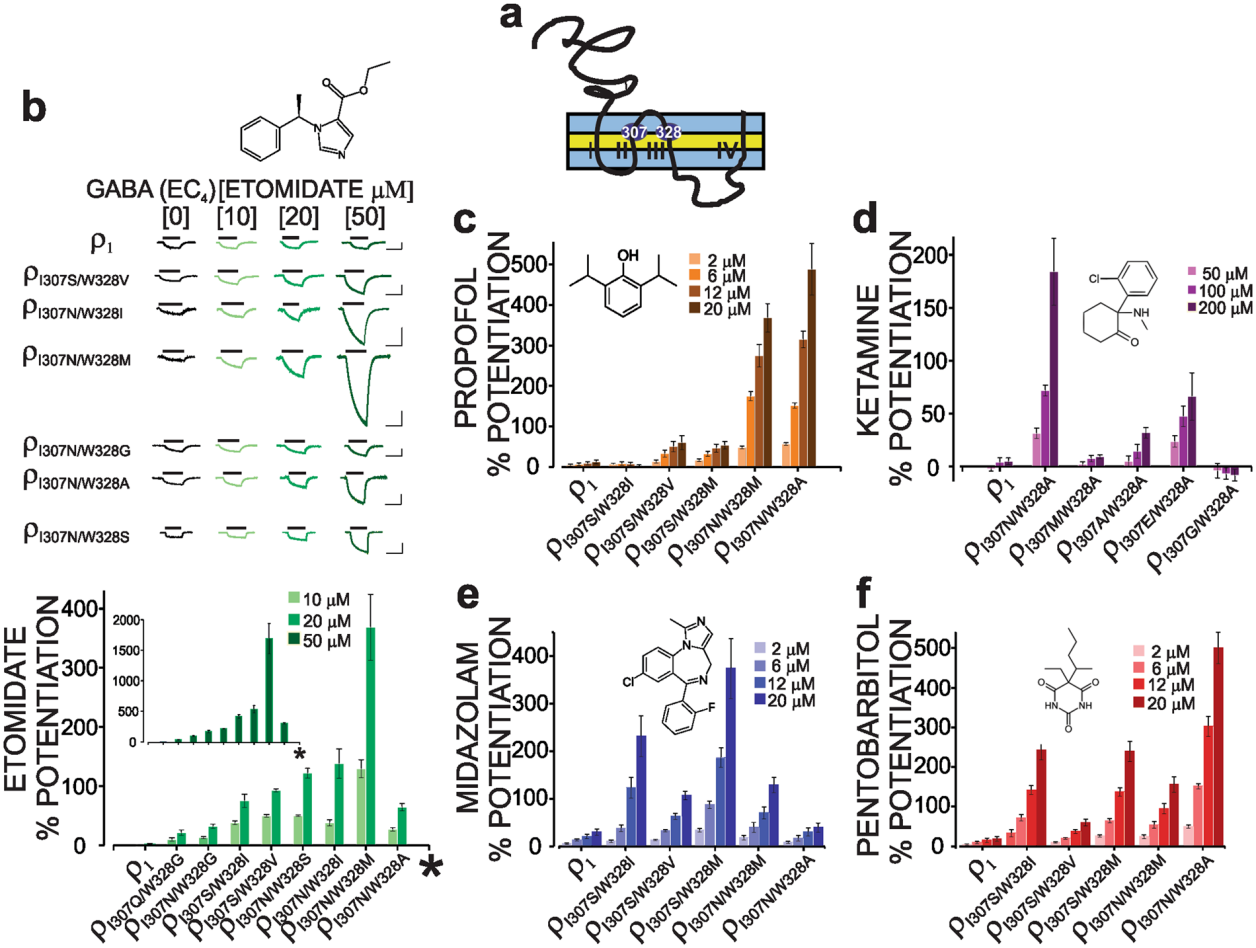
Mutations of the 307/328 residues confer sensitivity to the structurally distinct intravenous anaesthetics to the ρ_1_ receptor. (**a**) Schematic representation of the ρ_1_ subunit in the membrane bilayer. The positions of the 307 and 328 residues in the TM_2_ (II) and TM_3_ (III) are delineated. (**b**) Current traces and bar graphs represent the etomidate-dependent potentiation of the ρ_1_ 307/328 mutants. The lines above the current traces show the duration of the drug application. The vertical and horizontal bar scales denote 100 nA and 100 seconds, respectively. (**c**,**d**,**e**,**f**) The potentiation (as a percent increase) of the EC_4_ GABA currents in different ρ_1_ 307/328 mutants following the propofol-, ketamine-, midazolam-, and pentobarbital-dependent modulation.

The homo-oligomeric GABA_A_ ρ_1_ receptor is insensitive to the intravenous anaesthetics etomidate, propofol, ketamine, midazolam, and pentobarbital^56, 57^. To impart sensitivity to these structurally diverse classes of anaesthetics to the ρ_1_ receptor, we mutated the ρ_1_ subunit in TM_2_/TM_3_ at positions 307(Ile)/328(Trp). We then examined the responses of the resulting mutants to different concentrations of anaesthetics in the presence of their respective EC_4_ GABA (for EC_50_ values, see Table 1). Figure 1 shows the potentiating action of the GABA-evoked current from ρ_1_ 307/328 mutants in response to these structurally diverse intravenous anaesthetics. Several 307/328 double mutations of the ρ_1_ receptor conferred striking sensitivity to all the aforementioned anaesthetics (Fig. 1). The double mutants containing substitutions of Ile307 with Asn and Trp328 with Met or Ala exhibited a marked sensitivity to etomidate and propofol. Etomidate evoked 130 to 1700% potentiation at 10 to 50 µM of the ρ_I307N/W328M_ receptor (see Fig. 1b and Table 2 for the potentiation values). Propofol also markedly increased the GABA currents, resulting in approximately 50 to 500% potentiation of the ρ_I307N/W328M_ and ρ_I307N/W328A_ receptors (2 to 20 µM, Fig. 1c). We also assessed the sensitivity of a number of ρ_1_ 307/328 mutants to ketamine, which is a dissociative anaesthetic that acts mainly as an NMDA blocker and shows a positive modulatory action on the α_6_β_2/3_δ GABA_A_ receptor subtype^58, 59^. Regarding ρ_I307N/W328A_, ketamine at 50, 100, and 200 µM potentiated the GABA currents by approximately 30–200% (Fig. 1d). The benzodiazepine (midazolam and diazepam) and barbiturate (pentobarbital) classes of intravenous anaesthetics also significantly increased the GABA-induced currents in the 307/328 mutants (2 to 20 µM). The substitutions of Ile307 with Ser and then Ile307 with Asn produced the highest levels of potentiation with midazolam and pentobarbital, respectively (Fig. 1e and f). Overall, the propofol-, etomidate-, midazolam-, and pentobarbital-dependent modulation of the ρ_1_ 307/328 mutants occurred at clinically relevant concentrations. Thus, the 307/328 mutations conferred marked sensitivity to several classes of diverse anaesthetics including midazolam, pentobarbital, ketamine, propofol, and etomidate.

**Table 1:** Parameters determined from fitting the logistic equation to the data points of the GABA, I4AA, ZAPA, diazepam, and pentobarbital concentration-response relationships. All data are presented as the mean ± standard error (s.e.m.).

Subunit	EC_50_ (μM)	Slope	n
**GABA-dependent activation**			
ρ_1_	0.63 ± 0.03	2.55 ± 0.17	4
ρ_I307S/W328I_	0.06 ± 0.004	2.28 ± 0.17	4
ρ_I307S/W328V_	0.07 ± 0.003	2.45 ± 0.08	4
ρ_I307S/W328Y_	0.47 ± 0.01	3.04 ± 0.12	4
ρ_I307S/W328A_	1.00 ± 0.06	2.87 ± 0.09	7
ρ_I307N/W328A_	6.94 ± 0.61	1.47 ± 0.17	3
ρ_I307N/W328I_	0.14 ± 0.01	2.28 ± 0.11	3
ρ_I307N/W328M_	0.30 ± 0.02	3.08 ± 0.13	4
ρ_I307E/W328A_	4.17 ± 0.69	1.75 ± 0.12	4
ρ_I307A/W328A_	0.99 ± 0.06	2.66 ± 0.39	3
ρ_I307N/W328S_	26.30 ± 1.17	1.69 ± 0.06	4
ρ_I307G/W328A_	0.94 ± 0.04	3.14 ± 0.41	3
ρ_I307N/W328G_	0.48 ± 0.03	3.15 ± 0.08	4
ρ_I307M/W328A_	23.40 ± 3.72	1.40 ± 0.13	3
ρ_I307S/W328M_	0.10 ± 0.004	3.45 ± 0.08	4
ρ_I307Q/W328G_	0.47 ± 0.01	3.23 ± 0.05	5
ρ_I307N_	0.30 ± 0.01	2.61 ± 0.07	5
ρ_W328M_	1.57 ± 0.08	2.43 ± 0.05	4
**I4AA-dependent activation**			
ρ_1_	10.02 ± 0.57	1.14 ± 0.04	6
ρ_I307S/W328I_	0.27 ± 0.02	2.28 ± 0.17	5
ρ_I307S/W328V_	0.35 ± 0.03	2.22 ± 0.10	5
**ZAPA-dependent activation**			
ρ_I307S/W328I_	2.22 ± 0.09	1.74 ± 0.09	3
ρ_I307S/W328V_	4.01 ± 0.26	1.87 ± 0.14	5
**Diazepam-dependent activation**			
ρ_I307S/W328V_	102.34 ± 5.92	4.97 ± 0.82	5
ρ_I307S/W328V_:ρ_1_ (6:1)	114.55 ± 2.85	5.22 ± 0.37	7
ρ_I307S/W328V_:ρ_1_ (5:2)	119.62 ± 7.38	3.85 ± 0.28	7
**Pentobarbital-dependent activation**			
ρ_I307S/W328I_	180.77 ± 18.60	2.19 ± 0.09	5

**Table 2:** Potentiation values (% increase) of etomidate, propofol, midazolam, and pentobarbital in the presence of EC_4_ GABA in the ρ_1_ and ρ307/328 mutants. Pentobarbital- and diazepam-dependent potentiation of ~ EC_4_ GABA arising from ρ_1_ and different ratios of ρ_1_ mutant to wild-type. All data are presented as the mean ± standard error (s.e.m.).

Subunit					n
**Etomidate-dependent potentiation (%)**	**10 μM**	**20 μM**	**50 μM**		
ρ_1_	0.03 ± 0.28	1.05 ± 0.68	1.54 ± 1.77		5
ρ_I307Q/W328G_	9.58 ± 2.82	21.18 ± 4.63	39.88 ± 6.58		4
ρ_I307N/W328G_	13.06 ± 1.84	32.01 ± 3.62	100.75 ± 6.13		5
ρ_I307S/W328I_	37.90 ± 2.88	75.18 ± 11.39	176.27 ± 18.45		4
ρ_I307S/W328V_	49.55 ± 2.14	92.84 ± 2.60	223.80 ± 4.66		4
ρ_I307N/W328S_	49.81 ± 1.31	121.80 ± 8.20	424.70 ± 29.90		5
ρ_I307N/W328I_	37.97 ± 5.14	138.04 ± 24.77	541.96 ± 58.69		4
ρ_I307N/W328M_	129.37 ± 15.21	368.57 ± 55.79	1699.83 ± 248.05		4
ρ_I307N/W328A_	26.78 ± 2.91	64.29 ± 6.16	311.00 ± 9.55		5
**Propofol-dependent potentiation (%)**	**2 μM**	**6 μM**	**12 μM**	**20 μM**	
ρ_1_	4.68 ± 2.35	6.41 ± 3.04	8.71 ± 3.71	12.56 ± 4.83	5
ρ_I307S/W328I_	7.26 ± 1.97	8.31 ± 4.06	7.35 ± 4.34	1.87 ± 3.26	5
ρ_I307S/W328V_	13.34 ± 3.67	33.01 ± 8.88	50.50 ± 12.99	60.23 ± 17.23	5
ρ_I307S/W328M_	16.64 ± 3.00	32.34 ± 5.73	46.59 ± 9.88	53.74 ± 9.13	5
ρ_I307N/W328M_	48.34 ± 3.62	174.72 ± 11.19	274.60 ± 27.73	368.26 ± 35.08	6
ρ_I307N/W328A_	56.63 ± 2.93	151.69 ± 6.60	315.06 ± 20.58	488.10 ± 63.72	5
**Midazolam-dependent potentiation (%)**	**2 μM**	**6 μM**	**12 μM**	**20 μM**	
ρ_1_	5.74 ± 1.38	13.83 ± 2.00	21.13 ± 4.39	30.31 ± 6.67	6
ρ_I307S/W328I_	11.01 ± 2.23	38.30 ± 6.78	123.16 ± 21.64	231.55 ± 42.51	5
ρ_I307S/W328V_	13.69 ± 1.49	33.22 ± 2.61	63.10 ± 6.20	106.96 ± 8.90	5
ρ_I307S/W328M_	33.89 ± 3.34	87.79 ± 7.71	186.71 ± 20.44	374.12 ± 63.21	6
ρ_I307N/W328M_	18.39 ± 4.79	39.99 ± 10.70	70.54 ± 12.81	129.17 ± 15.90	4
ρ_I307N/W328A_	8.80 ± 2.29	16.89 ± 5.76	30.35 ± 8.42	39.80 ± 9.67	5
**Pentobarbital-dependent potentiation (%)**	**2 μM**	**6 μM**	**12 μM**	**20 μM**	
ρ_1_	4.49 ± 1.66	9.53 ± 2.80	15.41 ± 5.51	18.80 ± 6.02	7
ρ_I307S/W328I_	33.10 ± 8.77	72.18 ± 8.00	141.81 ± 10.87	242.69 ± 24.64	6
ρ_I307S/W328V_	9.73 ± 1.46	20.33 ± 2.07	37.41 ± 4.74	60.18 ± 8.67	5
ρ_I307S/W328M_	25.81 ± 2.62	64.87 ± 5.43	137.28 ± 10.66	239.60 ± 25.59	5
ρ_I307N/W328M_	24.15 ± 4.96	54.06 ± 8.48	95.14 ± 13.02	155.92 ± 19.61	9
ρ_I307N/W328A_	49.97 ± 4.28	151.42 ± 6.68	302.84 ± 25.55	500.60 ± 39.65	6
**Ketamine-dependent potentiation (%)**	**50 μM**	**100 μM**	**200 μM**		
ρ_1_	−2.08 ± 2.08	4.17 ± 4.17	5.05 ± 3.08		4
ρ_I307N/W328A_	31.40 ± 4.84	71.83 ± 5.13	184.00 ± 31.56		5
ρ_I307M/W328A_	2.27 ± 2.27	7.70 ± 3.09	9.36 ± 1.94		4
ρ_I307A/W328A_	4.99 ± 5.10	14.44 ± 6.48	32.02 ± 5.08		4
ρ_I307E/W328A_	23.69 ± 5.31	47.53 ± 9.72	66.27 ± 22.36		3
ρ_I307G/W328A_	-3.72 ± 6.83	-6.59 ± 5.37	-8.06 ± 5.50		4
**Pentobarbital-dependent potentiation (%)**	**20 μM**	**50 μM**	**100 μM**	**200 μM**	
ρ_1_	1.42 ± 1.31	5.23 ± 3.57			5
ρ_I307S/W328A_	226.92 ± 14.34	869.90 ± 88.52			6
22:1 (ρ_1_:ρ_I307S/W328A_)	10.65 ± 2.59	24.15 ± 5.71			5
5:2 (ρ_1_:ρ_I307S/W328A_)	30.93 ± 10.04	68.47 ± 10.72			5
4:3 (ρ_1_:ρ_I307S/W328A_)	51.97 ± 2.48	137.61 ± 8.77			5
3:4 (ρ_1_:ρ_I307S/W328A_)	78.65 ± 10.58	253.21 ± 34.83			5
2:5 (ρ_1_:ρ_I307S/W328A_)	113.34 ± 14.42	362.43 ± 57.53			5
ρ_1_	1.98 ± 0.72	3.88 ± 1.47	7.08 ± 2.48	9.44 ± 3.05	3
ρ_I307S/W328A_	260.08 ± 32.65	1195.71 ± 113.68	3369.08 ± 438.23	5038.85 ± 978.63	3
22:1 (ρ_1_:ρ_I307S/W328A_)	9.88 ± 0.31	18.68 ± 1.95	27.44 ± 3.15	36.45 ± 2.84	4
**Diazepam-dependent potentiation (%)**	**10 μM**	**30 μM**			
ρ_1_	10.43 ± 3.46	19.15 ± 4.33			3
ρ_I307S/W328Y_	99.79 ± 7.17	397.56 ± 29.27			5
22:1 (ρ_1_:ρ_I307S/W328Y_)	18.23 ± 4.92	42.17 ± 5.88			4
5:2 (ρ_1_:ρ_I307S/W328Y_)	19.25 ± 5.68	58.84 ± 8.03			5
4:3 (ρ_1_:ρ_I307S/W328Y_)	25.65 ± 5.60	93.03 ± 11.49			6
3:4 (ρ_1_:ρ_I307S/W328Y_)	40.47 ± 3.46	156.56 ± 9.16			6
2:5 (ρ_1_:ρ_I307S/W328Y_)	51.48 ± 5.34	256.95 ± 11.85			5

**Figure 2:**
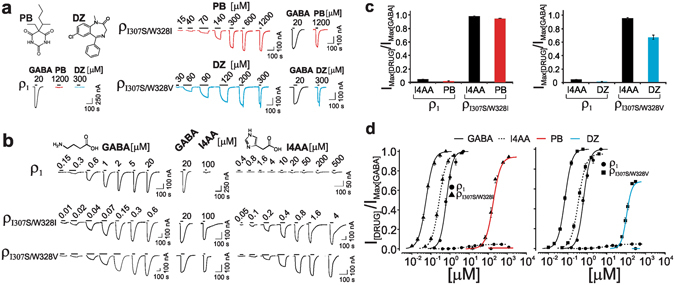
I4AA-, ZAPA-, pentobarbital-, and diazepam-dependent activation of ρ_1_ 307/328 mutants. (**a**) Pentobarbital (PB)- and diazepam (DZ)-induced current traces in the ρ_1_, ρ_I307S/W328I_, and ρ_I307S/W328V_ receptors. The lines above the current traces represent the duration of the drug application. (**b**) GABA- and I4AA-evoked current traces in the ρ_1_, ρ_I307S/W328I_, and ρ_I307S/W328V_ receptors. (**c**) The current maxima of I4AA, ZAPA, PB, and DZ relative to that elicited by GABA in the ρ_1_, ρ_I307S/W328I_, and ρ_I307S/W328V_ receptors. (**d**) The GABA, I4AA, PB and DZ concentration-response relationships in the ρ_1_, ρ_I307S/W328I_, and ρ_I307S/W328V_ receptors.

Pentobarbital and benzodiazepine diazepam were capable of directly activating the ρ_1_ 307/328 mutants at relatively higher concentrations. Figure 2a and d depict the current traces and the concentration-response relationships for pentobarbital and diazepam in the ρ_1_, ρ_I307S/W328I_, and ρ_I307S/W328V_ receptors. The wild-type ρ_1_ receptor was found to be insensitive to the direct action of these anaesthetics (Fig. 2). By contrast, both pentobarbital and diazepam directly activated the ρ_1_ 307/328 mutants in µM concentrations. Pentobarbital activated ρ_I307S/W328I_ with an EC_50_ of 181 ± 19 µM and a slope of 2.19 ± 0.09, while the corresponding numbers for the action of diazepam on ρ_I307S/W328V_ were 102.3 ± 5.9 µM and 5 ± 1. The maximum current amplitudes that were elicited by pentobarbital and diazepam relative to those elicited by GABA were approximately 0.94 in the ρ_I307S/W328I_ receptors and 0.68 in the ρ_I307S/W328V_ receptors (see Table 3 for the relative maximum amplitudes).

**Table 3:** The current maxima for pentobarbital, diazepam, I4AA, and ZAPA relative to that of GABA in the ρ_1_ and ρ307/328 mutants. All data are presented as the mean ± standard error (s.e.m.).

Subunit	Relative Maximum to GABA	n
**Pentobarbital**		
ρ_1_	0.02 ± 0.01	6
ρ_I307S/W328I_	0.94 ± 0.01	4
ρ_I307N_	0.08 ± 0.01	3
**Diazepam**		
ρ_1_	0.02 ± 0.01	7
ρ_I307S/W328V_	0.68 ± 0.03	9
**I4AA**		
ρ_1_	0.05 ± 0.003	17
ρ_I307S/W328I_	0.98 ± 0.01	5
ρ_I307S/W328V_	0.95 ± 0.02	6
ρ_I307N_	0.64 ± 0.03	4
ρ_I307S_	0.73 ± 0.02	4
ρ_W328M_	0.03 ± 0.01	4
**ZAPA**		
ρ_1_	0.04 ± 0.01	11
ρ_I307S/W328V_	0.98 ± 0.02	3
ρ_I307S/W328I_	0.98 ± 0.01	3

In summary, concomitant substitutions of ρ_Ile307_ with Asn or Ser and ρ_Trp328_ with Met or Ala imparted sensitivity to five structurally distinct anaesthetics to ρ_1_ receptors. In the anaesthetic-sensitive hetero-oligomeric α_1_β_2_γ_2_ GABA_A_ receptor, Asn and Ser were found at the corresponding TM_2_, while Met and Ala were found at the equivalent TM_3_ positions of the β_2_ and α_1_ subunits, respectively, thereby validating the use of the ρ_1_ receptor as a model system to study the mechanism of action of anaesthetics.

### Co-impartation of full efficacy to partial agonists

The GABA agonists imidazole 4-acetic acid (I4AA) and (Z)-3-[(aminoiminomethyl)thio] prop-2-enoic acid (ZAPA) are partial agonists of the ρ_1_ receptor but act as full agonists at the α_1_β_2_γ_2_ GABA_A_ receptors^60, 61^. We then examined the action of the GABA agonists I4AA and ZAPA in the ρ_1_ 307/328 mutants. Several 307/328 mutations, which have been shown here to confer sensitivity to anaesthetics to the ρ_1_ receptor, also converted the partial agonists I4AA and ZAPA into full agonists. Figure 2b and d show the GABA- and I4AA-induced current traces and the concentration-response relationships in the ρ_1_, ρ_I307S/W328I_, and ρ_I307S/W328V_ receptors. The maximal current amplitudes elicited by I4AA and ZAPA relative to those elicited by GABA in the wild-type ρ_1_ receptor were approximately 0.04. In marked contrast, both I4AA and ZAPA were full agonists in ρ_I307S/W328I_ or ρ_I307S/W328V_ (Both I4AA and ZAPA elicited maximal currents relative to that of GABA that were greater than 0.95, Table 3, Fig. 2c). In conclusion, the 307/328 mutations not only conferred sensitivity to diverse classes of anaesthetics but also imparted full efficacy to the partial agonists I4AA and ZAPA in the ρ_1_ receptor.

### Differential contributions of Ile307 and Trp328

We then asked whether the Ile307 and Trp328 mutations contribute differently to the conversion from partial to full GABA agonists and the impartation of anaesthetic sensitivity. To dissect the individual contribution of each mutation, the current maximal value of the I4AA relative to that of GABA was determined in the mutants ρ_I307S (or N)_ and ρ_W328M_. The single substitution mutation of Ile307 with Ser (ρ_I307S_) or Ile307 with Asn (ρ_I307N_) conferred nearly full efficacy to I4AA (a maximum-induced current of ~70% with respect to that of GABA). By contrast, I4AA was a partial agonist of ρ_W328M_ with an efficacy that was similar to that at the ρ_1_ receptor (Table 3). It has been shown previously that ρ_I307S_ displays a relatively low pentobarbital sensitivity at the potentiation level (with no apparent direct agonist action)^20^. By contrast, a substitution of Trp328 alone in the TM_3_ with any hydrophobic residue (e.g., ρ_W328M_) imparts a high sensitivity to pentobarbital to the ρ_1_ receptor at both the potentiation and direct activation levels (with maxima relative to that mediated by GABA of 10 to 20%)^19^. Thus, in the double mutant (e.g., ρ_I307S/W328I_), the Ile307 to Ser substitution contribute to the increasing efficacy, whereas the Trp328 mutation is key to conferring anaesthetic sensitivity to the ρ_1_ receptor.

### Distinct activation by GABA versus anaesthetics

**Figure 3:**
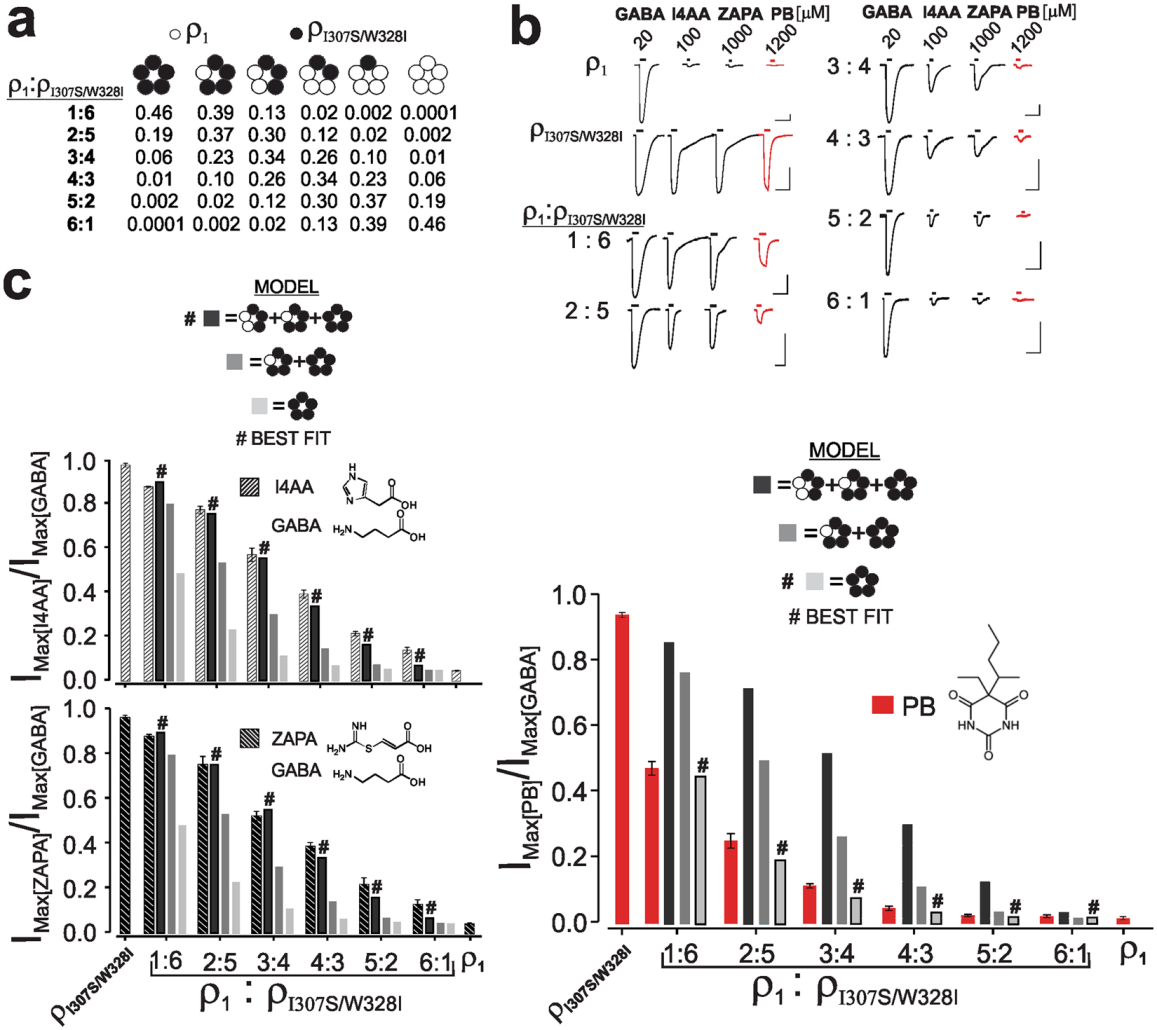
Variable co-expression of the ρ_1_ and 307/328 mutants reveals a distinct activation paradigm for GABA versus pentobarbital. (**a**) The predicted quantities of the receptor sub-populations resulting from the injection of different ratios of wild-type ρ_1_ to mutant cRNAs. (**b**) Current traces represent the maxima of GABA, I4AA, ZAPA, and pentobarbital (PB) in ρ_1_, ρ_I307S/W328I_, and different ratios of ρ_1_:ρ_I307S/W328I_. The lines above the current traces represent the duration of the drug application. The vertical and horizontal bar scales represent 100 nA and 100 seconds, respectively. (**c**) The current maxima of I4AA, ZAPA, and PB relative to that mediated by GABA in ρ_1_, ρ_I307S/W328I_, and different ratios of ρ_1_:ρ_I307S/W328I_. The three simulated models are shown in three shades of grey. The model representing the best fit is denoted by a hash # on the bar.

**Figure 4:**
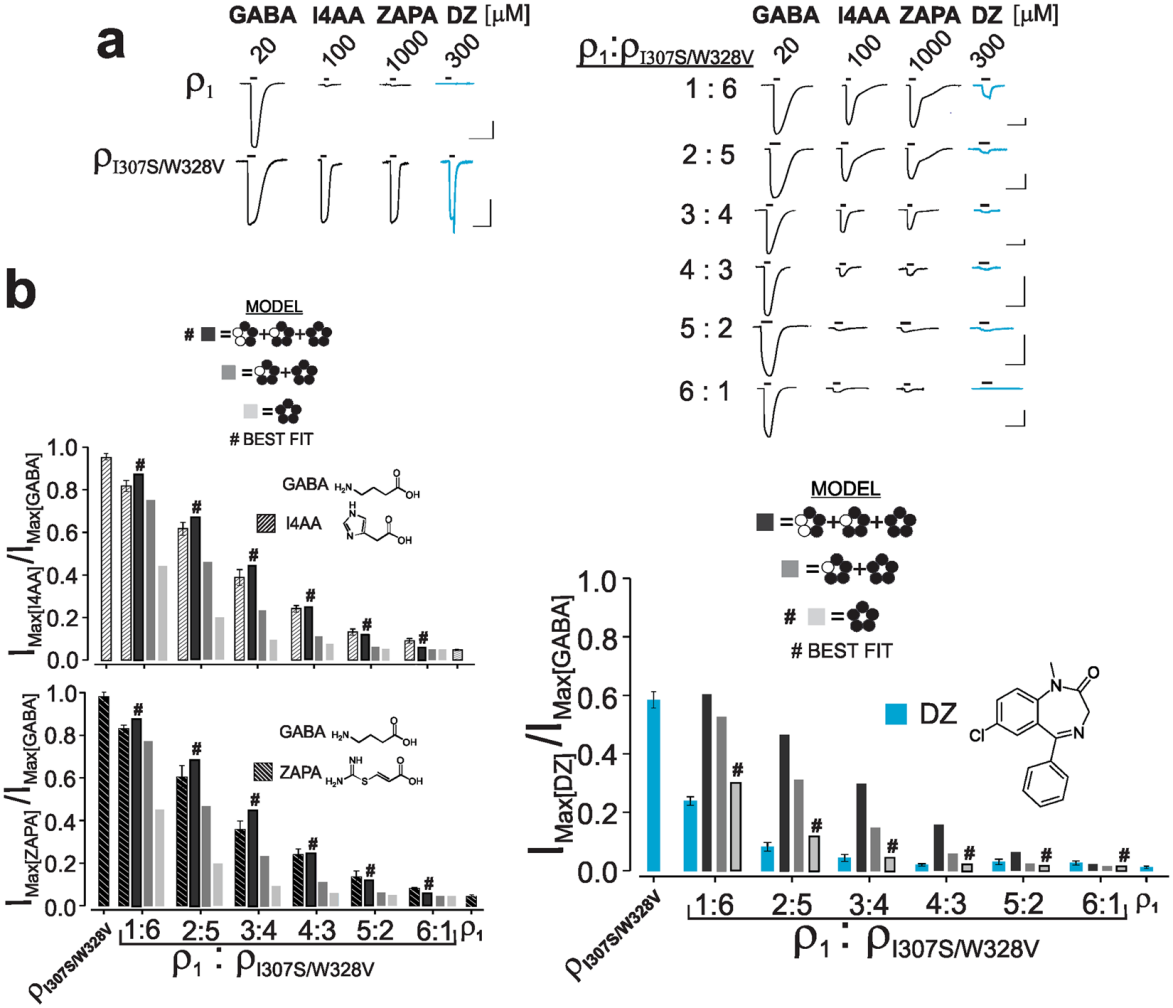
Variable co-expression of the ρ_1_ and 307/328 mutants demonstrates a distinct activation paradigm for GABA versus diazepam. (**a**) Current traces represent the maxima of GABA, I4AA, ZAPA, and diazepam (DZ) in ρ_1_, ρ_I307S/W328V_, and different ratios of ρ_1_:ρ_I307S/W328V_. The lines above the current traces represent the duration of the drug application. The vertical and horizontal bar scales represent 100 nA and 100 seconds, respectively. (**b**) The current maxima of I4AA, ZAPA, and DZ relative to that of GABA in ρ_1_, ρ_I307S/W328V_, and different ratios of ρ_1_:ρ_I307S/W328V_. The three simulated models are shown in three shades of grey. The model representing the best fit is denoted by a hash # on the bar.

We utilized the capacity of the ρ_1_ 307/328 mutations, which collectively impart full efficacy to otherwise partial GABA agonists and anaesthetic sensitivity, to compare the mechanism of activation of GABA agonists to that of anaesthetics. Using co-injection of cRNAs for the wild-type and the mutated ρ_1_ subunits at different ratios to express different ensembles of receptors containing five, four, three, two, one, or zero mutated subunits, we attempted to identify the number of mutated subunits that is sufficient 1) to confer full efficacy to otherwise partial GABA agonists and 2) impart anaesthetic sensitivity. Prior to the experiments, the maximal GABA-induced current amplitudes of the key mutants (ρ_I307S/W328I_ and ρ_I307S/W328V_) relative to that of wild-type were first examined following equivalent injections of each mutant versus wild-type cRNAs (see Materials and Methods). These experiments yielded maximal GABA-induced currents in ρ_I307S/W328I_ and ρ_I307S/W328V_ relative to that for wild-type ρ_1_ of 0.93 and 0.43, respectively (Table 4). Thus, ρ_I307S/W328I_ exhibited a maximal GABA-induced current that was nearly equal to that of the ρ_1_ receptor, while for the ρ_I307S/W328V_, this value was approximately half of that of the ρ_1_ receptor. Then, the cRNAs of ρ_1_ and ρ_I307S/W328I_ or ρ_1_ and ρ_I307S/W328V_ at ratios of 1:6, 2:5, 3:4, 4:3, 5:2, and 6:1 (ρ_1_: ρ_307/328 mutants_) were co-injected to express distinct ensembles of the following six subpopulations of receptors: homo-oligomers of wild-type and mutant subunits and hetero-oligomers containing one, two, three, and four mutated subunit(s). For the controls, the cRNAs of ρ_1_, ρ_I307S/W328I_, or ρ_I307S/W328V_ were also injected individually. In each injected oocyte, we then determined the maximal currents evoked by GABA, I4AA, ZAPA, and pentobarbital after injections of different ratios of ρ_1_: ρ_I307S/W328I_, ρ_1_ and ρ_I307S/W328I_; we further determined the maxima of GABA, I4AA, ZAPA, and diazepam with varying ratios of ρ_1_:ρ_I307S/W328V_, ρ_1_, and ρ_I307S/W328V_. The maximal currents that were evoked by I4AA, ZAPA, and the anaesthetics were then normalized to their respective GABA maximal current values (see Materials and Methods). The averages of the relative current maxima (to that elicited by GABA) with I4AA, ZAPA, or pentobarbital with ρ_1_; ρ_I307S/W328I_; 1:6, 2:5, 3:4, 4:3, 5:2, and 6:1 cRNA ratios of ρ_1_: ρ_I307S/W328I_ are presented in Fig. 3 whereas the averages of the relative current maxima (to that of GABA) with I4AA, ZAPA, or diazepam with ρ_1_; ρ_I307S/W328V_; and varying ratios of ρ_1_: ρ_I307S/W328V_ are shown in Fig. 4. With increases in the ratio of the wild-type to mutated cRNAs, a progressive reduction in the relative current maxima (to that elicited by GABA) for I4AA, ZAPA, pentobarbital, or diazepam was discernible, but the degree of the overall decline at each ratio was markedly greater with the anaesthetics (diazepam or pentobarbital) than the GABA agonists (I4AA or ZAPA). For example, for the 1:6 ratio of ρ_1_: ρ_I307S/W328I_ cRNAs, the relative current maxima (to that mediated by GABA) decreased to approximately 0.87 with both GABA agonists, i.e., I4AA and ZAPA (from 0.98 in the homo-oligomeric ρ_I307S/W328I_); the corresponding values of the 1:6 ratio of ρ_1_: ρ_I307S/W328V_ declined to 0.79 and 0.82 with I4AA and ZAPA, respectively (from ~0.95 in ρ_I307S/W328V_, Supplementary Information-Datasets). By contrast, the relative current maxima of pentobarbital or diazepam (to that elicited by GABA) at 1:6 (ρ_1_: ρ_307/328 mutants_) exhibited a significantly greater decline compared to that of I4AA or ZAPA, thereby diminishing the corresponding value of the 1:6 ratio of ρ_1_: ρ_I307S/W328I_ to 0.47 with pentobarbital (from 0.94 in ρ_I307S/W328I_) and of the 1:6 ratio of ρ_1_:ρ_I307S/W328V_ to 0.23 with diazepam (from 0.68 in ρ_I307S/W328V_). The differential reductions in the relative current maxima (with respect to that induced by GABA) between the GABA agonists and the anaesthetics continued after increasing the ratio of the wild-type to the mutated cRNAs, showing a greater prominence with diazepam. The decline in the relative current maximum (to that of GABA) with diazepam was markedly greater than that with pentobarbital across the different ratios, which may be due to 1) the lesser maximum current with diazepam (to that mediated by GABA) in the homo-oligomeric ρ_I307S/W328V_ than that with pentobarbital in ρ_I307S/W328I_ and 2) the lower GABA maximal current (based on maximal GABA-induced current for ρ_I307S/W328V_ relative to that for wild-type, at equivalent cRNA injection) of ρ_I307S/W328V_ compared to that of the wild-type (Table 3).

**Table 4:** The GABA-induced maximal current of key ρ_307/328_ mutants (used in the mixing experiments) relative to that of ρ_1_ at equivalent cRNA injections. All data are presented as the mean ± standard error (s.e.m.).

Subunit	GABA-evoked maximal current(nA) mutant/wild-type at equal injections	Mutant expression relative to wild-type ρ_1_
ρ_I307S/W328I_	276 ± 28 (n = 23)/296 ± 32 (n = 19)	276/296 = 0.93
ρ_I307S/W328V_	128 ± 15 (n = 25)/296 ± 32 (n = 19)	128/296 = 0.43
ρ_I307S/W328Y_	334 ± 16 (n = 20)/556 ± 43 (n = 14)	334/556 = 0.60
ρ_I307S/W328A_	1061 ± 26 (n = 11)/903 ± 35 (n = 14)	1061/903 = 1.18

We used a binomial equation to determine the relative quantities of the receptor sub-populations that contained five, four, three, two, one, or zero mutated subunits at each ratio and assumed an equivalent assembly of wild-type and mutated subunits (Fig. 3a, Supplementary Information-Datasets). Then, using an iterative process, we conducted simulation studies to determine the likelihood of contribution of each sub-population of receptor(s) in the ensemble toward the total response to I4AA, ZAPA, or the anaesthetics. In the subpopulation ensembles at each ratio, the experimentally determined values were utilized for the homo-oligomers of the wild-type or mutated receptors, while, depending on the model, all (homo-oligomeric mutant-like activity) or none of the weight (wild-type-like activity) was assigned to the hetero-oligomeric receptors that contained four, three, two, or one mutated subunits with unknown activity. Three different models were tested. In the first model, the contribution of only the subpopulation of homo-oligomeric mutant receptors with all the weight activity (homo-oligomeric mutant-like activity) given to the overall current was considered; the remainder of the sub-populations was speculated to have wild-type-like activity (close to zero). In the second model, two receptor sub-populations in the ensemble were simulated to have all the weight mutant-like activity, including the homo-oligomer of the mutant and the hetero-oligomer with the four mutated subunits. The remainder of the four subpopulations was presumed to have wild-type like activity. Finally, in the third model, three subpopulations of receptors containing five, four, and three mutated subunits were assumed to exhibit mutant-like activity, while the remaining three subpopulations were believed to exhibit wild-type-like activity. In the simulation of the total activity at each ratio, the known (homo-oligomer) values and the presumed (hetero-oligomer) values for each receptor sub-population were multiplied by the corresponding sub-population fraction that was present in the ensemble (determined using a binomial equation). The resulting values were then summed (For details regarding the simulation procedures, see Methods and Supplementary Information-Datasets). In comparison to the wild-type, all simulations were corrected for the lower maxima current (relative to that mediated by GABA) of diazepam or pentobarbital in the homo-oligomeric ρ_I307S/W328V_ or ρ_I307S/W328I_, as well as the lower GABA maximal current of ρ_I307S/W328V_ (based on maximal GABA-induced current for mutant relative to that for wild-type, at equivalent cRNA injection). The conclusions were unaffected even if no corrections for the differences in the GABA-induced maxima were included in the simulation steps for ρ_I307S/W328V_ (see Supplementary Information-Datasets). Figures 3 and 4 show the three simulations for the ρ_1_:ρ_I307S/W328I_ and ρ_1_:ρ_I307S/W328V_ co-expression studies (in the form of bars and different shades of grey). A comparison of the data points with the three different simulations at each ratio demonstrated that the summation of the contributions of the receptors containing three or more mutated subunits (i.e., the summation of the receptors containing five, four, and three mutated subunits) with mutant-like activity best matched the experimental data of the GABA agonists I4AA and ZAPA (denoted by a hash # on the bar, Figs 3c and 4b). In striking contrast, the model simulation that represented only the contribution of the homo-oligomer of the 307/328 mutant subunits with mutant-level activity (only the receptor sub-population of five mutated subunits) corresponded to the experimental data of pentobarbital (Fig. 3c, denoted by a hash #) and diazepam (Fig. 4b, denoted by a hash #).

Then, we constructed diazepam concentration-response relationships for the 1:6 and 2:5 ratios from the ρ_1_: ρ_I307S/W328V_ experiments. These experiments were carried out to determine whether the diazepam-induced current arises solely from a single sub-population of receptors (ρ_I307S/W328V_) or a mixture of homo- and hetero-oligomeric receptor-channels (with different EC_50_s and slopes) in the co-expressional experiments. The derived EC_50_ and Hill coefficient in these experiments were nearly identical to the corresponding values in the ρ_I307S/W328V_ receptor (Table 1), indicating that the diazepam-induced current observed in the experiment using the 6:1 or 2:5 ratios of ρ_1_: ρ_I307S/W328V_ cRNAs arose mainly from the sub-population of the homo-oligomeric ρ_I307S/W328V_.

In summary, our data indicate that GABA and anaesthetics act via distinct mechanisms in terms of the number of mutated subunits that are necessary for direct activation; three 307/328 mutated subunits are sufficient for the GABA-dependent action, while the corresponding mutations must be present in all five subunits for the anaesthetic-dependent activation to transpire.

### A single mutated subunit confers anaesthetic-dependent potentiation of GABA currents

**Figure 5:**
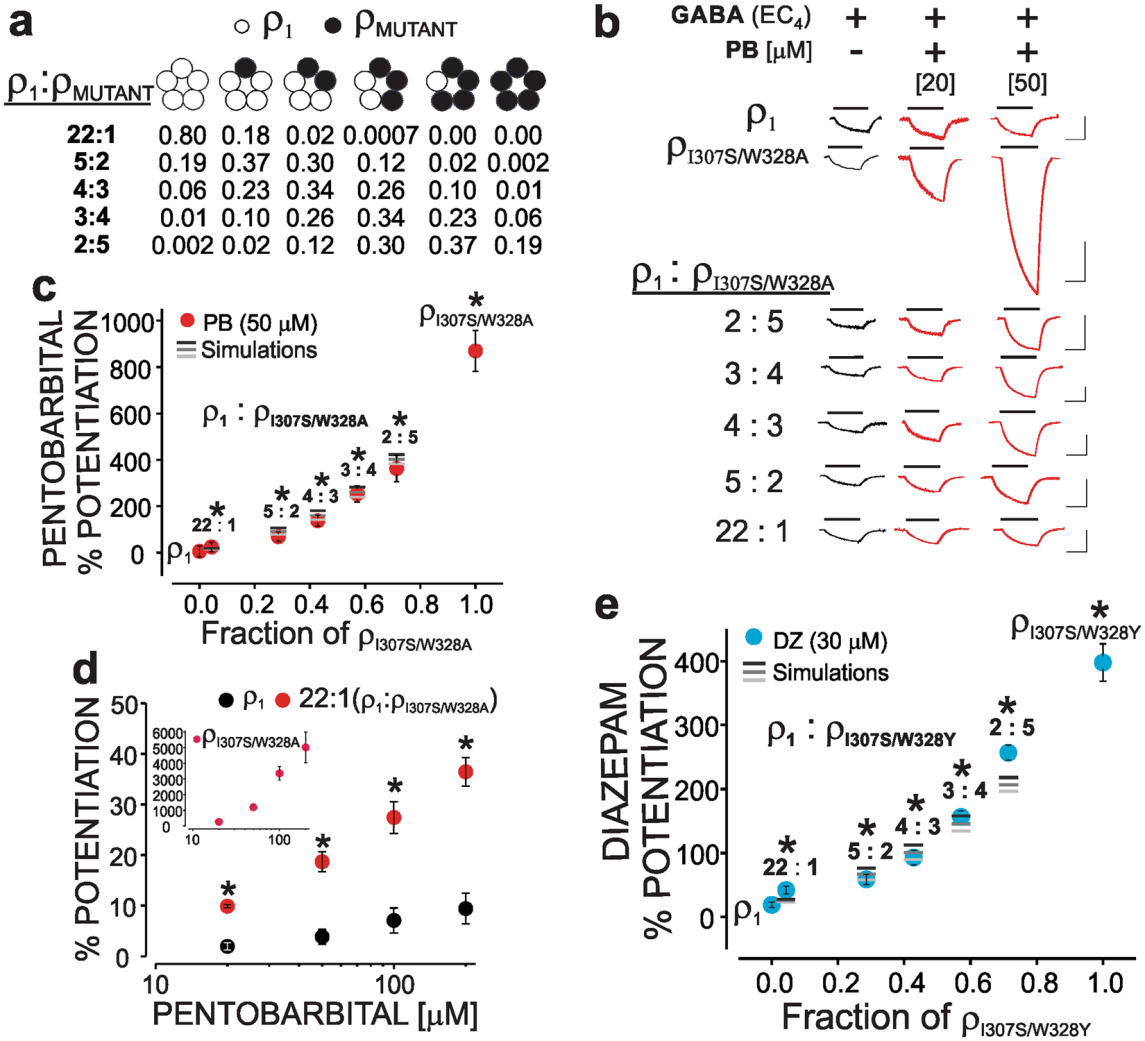
Hetero-oligomeric ρ_1_ receptors containing a single mutated subunit confer anaesthetic potentiation. (**a**) The predicted quantities of the receptor sub-populations expected from the co-expression of different ratios of wild-type to mutant cRNA. (**b**) Current traces elicited by EC_4_ GABA and EC_4_ GABA plus 20 or 50 µM PB for ρ_1_, ρ_I307S/W328A_, and different ratios of ρ_1_:ρ_I307S/W328A_. The lines above the current traces represent the duration of the drug application. The vertical and horizontal bar scales denote 50 nA and 100 seconds, respectively. (**c**) PB-dependent potentiation of EC_4_ GABA for ρ_1_, ρ_I307S/W328A_, and different ratios of ρ_1_:ρ_I307S/W328A_. (**d**) Potentiation of the EC_4_ GABA by 20, 50, 100, and 200 µM PB for ρ_1_, and 22:1 ratio of ρ_1_:ρ_I307S/W328A_ as well as ρ_I307S/W328A_ (inset). (**e**) Potentiation of the EC_4_ GABA by 30 µM DZ for ρ_1_, and different ratios of ρ_1_:ρ_I307S/W328Y_. The three shades of grey horizontal lines in c and e are simulated models for the potentiation experiments. The differences in the potentiation levels between the different ratios of the ρ_1_:mutant and ρ_1_ are statistically significant (p < 0.05).

We then examined the mechanism underlying the anaesthetic-dependent modulation of the GABA current by deciphering the minimal number of mutated subunits that are necessary to confer potentiation. The co-expression of cRNAs for the wild-type with ρ_I307S/W328Y_ or ρ_I307S/W328A_ at different ratios were used to determine the mechanism underlying the anaesthetic-dependent potentiation at the subunit level. The ρ_I307S/W328Y_ receptor showed a high sensitivity to diazepam, while the ρ_I307S/W328A_ receptor exhibited a marked sensitivity to pentobarbital in potentiation action (see Tables 1, 3, and 4). At equivalent cRNA injection, ρ_I307S/W328A_ exhibited a maximal GABA-induced current that was nearly equal to that of the ρ_1_ receptor, while for the ρ_I307S/W328Y_, this value was approximately 0.6 of that of the wild-type (Table 4). The GABA concentration-response relationship was constructed for ρ_I307S/W328A_ and ρ_I307S/W328Y_. These experiments demonstrated that the ρ_I307S/W328A_ and ρ_I307S/W328Y_ mutants had GABA EC_50_s that were similar to those of the wildtype (~1 and 0.5, respectively, compared to 0.6 µM in the wild type). This finding was an important consideration since the degree of the potentiation magnitude is highly dependent on the relative GABA-induced activity of the receptor-channel^22^. To determine the minimal number of mutated subunits that are necessary to confer potentiation, the cRNAs of ρ_1_ and ρ_I307S/W328Y_ or ρ_1_ and ρ_I307S/W328A_ were co-injected at ratios of 22:1, 5:2, 4:3, 3:4, and 2:5 (ρ_1_: ρ_307/328 mutant_). In the presence of the approximate EC_4_ GABA, the extents of the diazepam- (30 µM, for ρ_I307S/W328Y_) and pentobarbital- (20 and 50 µM, for ρ_I307S/W328A_) dependent potentiation were then determined at each ratio. Figure 5 shows the pentobarbital (ρ_I307S/W328A_)- and diazepam (ρ_I307S/W328Y_)-dependent potentiation levels of ρ_1_, ρ_I307S/W328A_, ρ_I307S/W328Y_, as well as of different ratios of ρ_1_: ρ_I307S/W328A_ and ρ_1_: ρ_I307S/W328Y_. In the presence of the EC_4_ GABA, pentobarbital (50 µM) caused only a minuscule change in the GABA currents arising from the ρ_1_ receptor but increased the corresponding GABA current of ρ_I307S/W328A_ by 870 ± 89% (Table 2). At the 22:1 ratio (wild-type:mutant), assuming an equal assembly of wild-type and mutant subunits, the binomial calculations predicted that 80% of the constituted receptors in the ensemble were wild-type, while the remainder were comprised of primarily hetero-oligomeric receptors with only a single mutated subunit (four wild-type, Fig. 5a). At the 22:1 ratio of ρ_1_: ρ_I307S/W328A_, pentobarbital (20, 50, 100, or 200 µM) produced a potentiation that was significantly greater than that in the wild-type (Fig. 5c and d; statistically greater than wild-type, p < 0.05, Supplementary Information-Datasets). In the diazepam-dependent modulation, there was also a statistically greater potentiation compared to that in the wild-type in the experiments corresponding to the 22:1 ratio of ρ_1_: ρ_I307S/W328Y_ (Supplementary Information-Datasets). Thus, in contrast to the direct receptor activation by diazepam or pentobarbital, the modulatory properties of the anaesthetics can be imparted to the receptor sub-population containing a single mutated subunit.

To study the mechanism underlying the anaesthetic-dependent modulation, we constructed models to carry out potentiation simulations at each ratio. For these calculations, we used the experimentally determined potentiation values for the subpopulation of receptors corresponding to the homo-oligomers of the wild-type or mutant subunits. However, the values of the potentiation magnitude arising from hetero-oligomeric receptors containing one, two, three, or four mutated subunit(s) were unknown and were therefore estimated by reducing the known potentiation values of the mutated homo-oligomers by ~0.5^n^ (0.47^n^, 0.5^n^, and 0.53^n^ for pentobarbital; 0.57^n^, 0.6^n^, and 0.63^n^ for diazepam), where n represents the number of the wild-type subunits in the pentamer. The numbers (~0.5^n^) used in these simulations to estimate the potentiation values of the hetero-oligomeric channels at the tested concentrations of the anaesthetics were derived using an iterative process. The total potentiation simulations at each ratio, as shown in Fig. 5, were then calculated by multiplying the known (for homo-oligomers) and the presumed (for hetero-oligomers) potentiation values by the corresponding fraction of the subpopulations that were present in each ensemble (determined using the binomial equation) followed by summing the resulting values (Supplementary Information-Datasets). Figure 5 depicts the three simulations for each co-expression at different ratios of wild-type:mutant experiments (in the form of horizontal lines, different shades of grey). For each ratio, the simulation numbers corresponded closely to the data points of the pentobarbital- or diazepam-dependent potentiation (Fig. 5c and e). An examination of the simulated potentiation values of each receptor sub-population reveals that the sequential replacement of each wild-type subunit with a mutant subunit in the pentamer did not appear to increase the potentiation levels synergistically in the tested concentration range of the anaesthetics. For example, a single hetero-oligomeric receptor with two mutated subunits (of ρ_I307S/W328A_) generates a potentiation level (e.g., 0.5^3(# of wild-type subunits)^*870%) that is nearly equal to the sum of the potentiation values of two receptors each having a single mutated subunit (e.g., 0.5^4(# of wild-type subunits)^*870% + 0.5^4^*870% = 2*0.5^4^*870% = 1*0.5^3^*870%).

Collectively, these studies demonstrate that the magnitude of the potentiation declines sequentially along with the reduction in the number of mutated subunits in the pentamer. Importantly, receptors that contain even a single mutated subunit are sensitive to the potentiation action of the anaesthetics.

## Discussion

Using coexpression of cRNAs for the wild-type and mutated (307/328) ρ_1_ subunits at different ratios, we demonstrate that the number of anaesthetic-sensitive ρ_1_ subunits crucial for imparting full efficacy to the partial GABA agonists in the pentamer is three, while the number needed to confer anaesthetic sensitivity at the level of direct activation is five. Importantly, the number of anaesthetic-sensitive ρ_1_ subunits needed to convey potentiation by the anaesthetics is one.

Mutations in the key residues ρ_Ile307_ and ρ_Trp328_ play distinctive roles in the co-impartation of the full efficacy to the partial GABA agonists (I4AA) and anaesthetic sensitivity to the ρ_1_ receptor. Both Ile307 and Trp328 are located at the hydrophobic/hydrophilic interface in the upper leaflet of the membrane bilayer; however, the Trp side chain not only constitutes the largest volume among all amino acids, but it also has the potential to anchor the TM_3_ polypeptide to the membrane interface. Mutations in ρ_Trp328_ could dislodge the TM_3_ from the membrane interface and create a void, thus exposing the delicate gating components to anaesthetic action. However, the substitution of the highly hydrophobic ρ_Ile307_ with the hydrophilic Ser can shift the gating component, which is located in the TM_2_, closer to the hydrophilic upper leaflet, hence contributing to an increase in the efficacy of the GABA agonists (and allosteric agonists). Collectively, the double 307/328 mutations may create novel relaxed state(s) with relatively reduced free energy levels of activation^44^, in which access to or efficient alignment with the molecular actions of anaesthetics is probable.

**Figure 6:**
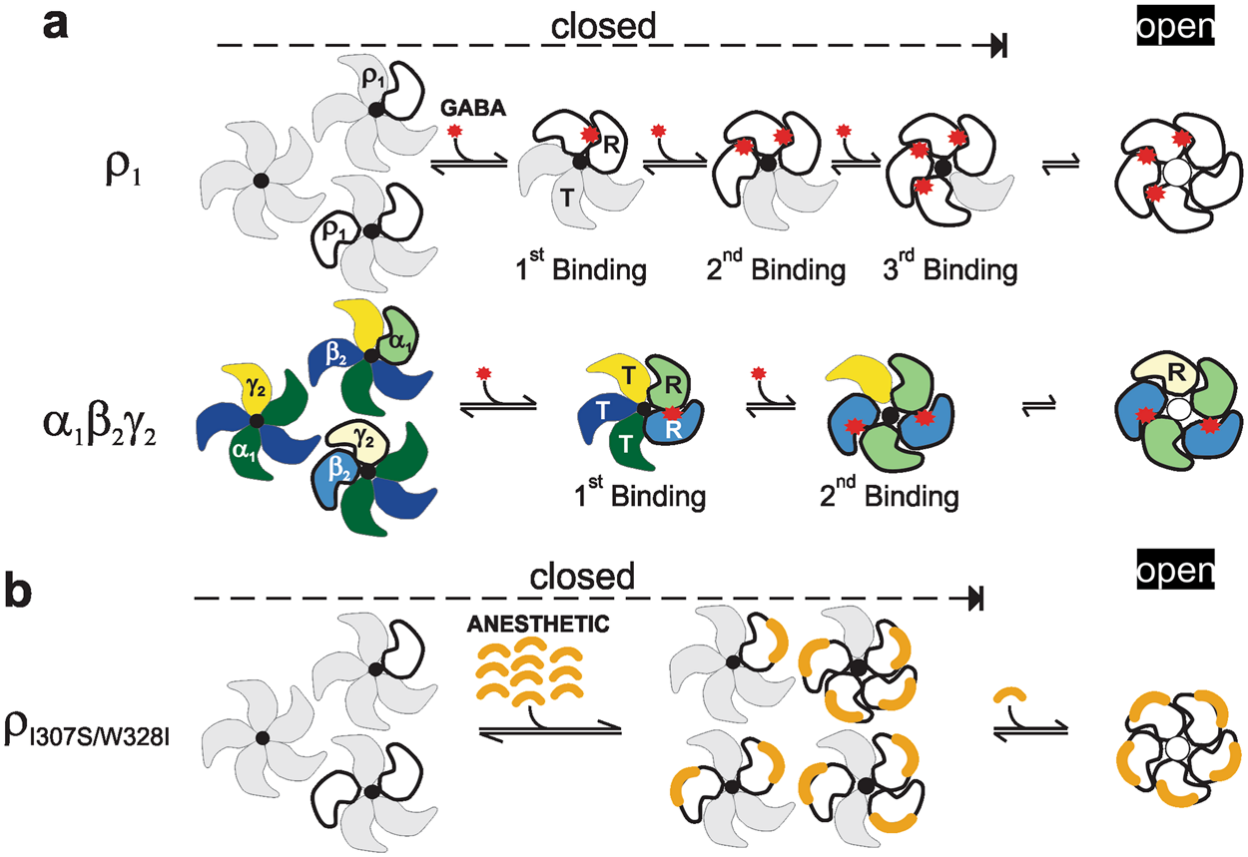
A model of GABA- versus anaesthetic-dependent activation. (**a**) A model of the GABA-dependent activation of α_1_β_2_γ_2_ compared to that of the ρ_1_ GABA_A_ receptors. T and R represent tense and relaxed states, respectively. Note that in this model, a single GABA binding can stabilize two subunits into a relaxed state and cast a more widespread effect on the overall structure. For the α_1_β_2_γ_2_ receptor, the intermittent binding of two GABA molecules can stabilize four subunits into a relaxed state, while for the ρ_1_ receptor, three consecutive GABA binding events (three GABA molecules) are needed to achieve the same task. (**b**) Represents the distinct model of the anaesthetic-dependent activation of the mutated ρ_1_ receptors. In the presented model, the anaesthetics produce a local and limited effect on the state of the subunits.

Our key finding is that the activation of GABA_A_ receptors by GABA via orthosteric sites compared to that by anaesthetics via allosteric sites requires numerically distinct subunit level rearrangements. In the GABA-dependent activation mode, the number of GABA binding steps (at the orthosteric sites) needed to open the channel differs between the homo-oligomeric ρ_1_ and the hetero-oligomeric α_1_β_2_γ_2_ receptors^3, 49–52, 62, 63^. It is currently well-established that for the ρ_1_ receptor, the required number of GABA bindings to open the channel is three (one per subunit, with five total subunits)^50, 51^, while for the α_1_β_2_γ_2_ receptor, the required number of GABA bindings is only two^49, 64^ (one per β-α subunits; out of five). This raises the following question: what are the underlying structural and mechanical differences underlying the lower efficiency that is observed in the GABA-dependent activation of ρ_1_ compared to that of α_1_β_2_γ_2_ receptor? The α_1_β_2_γ_2_ receptor exhibit fixed stoichiometry with two non-equivalent, but predetermined, GABA binding sites intermittingly positioned at the β-α interface of the pentamer (See Fig. 6), which is similar to the homologous hetero-oligomeric nicotinic acetylcholine receptor^65^. GABA agonists bind to the extracellular domain in the interface of the two subunits with an asymmetrical geometry, presumably via a strong electrostatic bonds^66, 67^. Thus, the binding of GABA to the higher affinity site may impart structural perturbation to the two subunits, leading to a facilitation of subsequent secondary binding in the α_1_β_2_γ_2_ receptor. Consequently, the sequential but intermittent bindings of two GABA molecules at the orthosteric sites have the capacity to impact four subunits, thus rendering them into the relaxed state. In comparison, for the ρ_1_ receptor, the first binding can occur randomly at any of the five potential GABA binding sites at the interface, potentially transforming two subunits into their relaxed states. This first binding then cooperatively facilitates the second consecutive binding at the adjacent subunit. However, the perturbation (stabilization) caused by the secondary binding to the ρ_1_ receptor may transmit to only three subunits. Therefore, to complete the stabilization of the four subunits into their relaxed states, GABA binding to a third consecutive site is needed (see the presented model in Fig. 6). Therefore, in a model where rendering four subunits into the relaxed state via the orthosteric sites dictates an open configuration, the number of GABA molecules required for the α_1_β_2_γ_2_ receptor binding is two, while for the ρ_1_ receptor, the number required is three. Thus, through efficient inter-subunit action (location) and the presumed strong nature of its binding force, GABA can exert a relatively global action on the structure of the receptor-channel^68^. In contrast to GABA action, our data support the notion that anaesthetics act locally and transmit a more limited force on the stabilization of the channel in the open configuration. The following three findings support the local effects of anaesthetics: 1) Anaesthetic molecules act allosterically in the channel in the transmembrane medium close to the gating component likely through a weak hydrophobic interaction. 2) The five-subunit (the entire pentamer) requirement to confer anaesthetic-dependent direct activation indicates the weak nature of the transduction in opening the channel. 3) A single anaesthetic-sensitive subunit, paradoxically, confers an anaesthetic-dependent potentiation, but the addition of each mutated subunit does not appear to increase the potentiation levels synergistically. How can one explain the differences in the requirement for activation versus modulation (all 5 subunits versus 1 subunit)? In the modulatory mode, in a model in which three sequential GABA binding events stabilize the channel in the open state, the anaesthetic-dependent activation of a single subunit needs to enhance the binding of GABA to the receptor only in the first binding step, thus increasing the efficiency of the subsequent GABA bindings and the eventual channel opening. Collectively, these findings indicate that, unlike GABA, the force of anaesthetics does not appear to propagate to the neighbouring subunits, is limited in its scope and poses only a local effect on the channel.

The interaction between the GABA agonist and the orthosteric sites required to open the channel has been evolutionarily optimized through precise/specific positioning of the GABA binding sites, the tuning of the inter-subunit dynamics, and the facilitation of the transduction/stabilization processes. Anaesthetic effects, by contrast, appear to be more generic, their site of action is not as fine-tuned, and their transduction/stabilization is not as enhanced. In the evolutionary ladder of ligand-gated ion channels, the hetero-oligomeric receptors (e.g., α_1_β_2_γ_2_) evolved more recently^69^. The fact that two versus three GABA molecules are needed to bind the receptor to open the hetero- versus homo-oligomer of GABA_A_ receptors suggests that the optimization in terms of the tuning of the inter-subunit dynamic and the facilitation of the transduction/stabilization processes has resulted in a binding/opening process in the hetero-oligomeric α_1_β_2_γ_2_ that is more efficient than that in the ρ_1_ receptor. Thus, the difference in the α_1_β_2_γ_2_ receptor versus the ρ_1_ receptor predicts that in the allosteric-dependent activation by anaesthetics, the number of subunits required to bind (sense) the anaesthetic may be lower in the α_1_β_2_γ_2_ receptors relative to that in the ρ_1_ GABA_A_ receptors.

Our findings demonstrate that, in comparison to GABA, anaesthetic molecules not only use a different site of action but also exhibit a different activation paradigm to maintain the channel in the open state. Thus, allosteric molecules such as anaesthetics can modulate GABA-gated ion channels in a dynamically distinct fashion.

## Methods

### Oocyte preparation and electrophysiology

The oocyte isolation, site-directed mutagenesis, complementary RNA (cRNA) synthesis, cRNA injection into the oocyte, the drug perfusion system, and the oocyte electrophysiology have been previously described^22, 58^. The quality of the cRNAs was determined by electrophoresis of set dilutions of the cRNA on a 1% formaldehyde-containing agarose gel. The amount of cRNA was first determined and matched by interpolation of lanes containing different dilutions of the cRNA and then quantified spectrophotometrically. Following the injection, the oocytes were incubated in a solution containing the following (in mM): 5 HEPES, 82.5 NaCl, 2.5 KCl, 1 CaCl_2_, 1 MgCl_2_, 1 Na_2_HPO_4_, and 2.5 Na pyruvate, with the pH adjusted to 7.5 with NaOH. This mixture was supplemented with 50 U/ml penicillin, 50 µg/ml streptomycin, and 2% horse serum. The oocytes were maintained at 14 °C. The recording solution (OR_2_) contained the following (in mM): 5 HEPES, 92.5 NaCl, 2.5 KCl, 1 CaCl_2_, and 1 MgCl_2_, with the pH adjusted to 7.5 with NaOH. All methods relating to animal procedures were approved by Animal Care and Use Committee of University of South Florida, and were carried out in accordance with Guidelines of the National Institute of Health for the Use of Laboratory Animals.

### Comparison of the wild-type and mutant expression levels

To measure the expression levels of the key mutant subunits (ρ_I307S/W328I_, ρ_I307S/W328V_, ρ_I307S/W328Y_, and ρ_I307S/W328A_) relative to those of the wild-type subunits, the cRNAs of the wild-type or mutant ρ_1_ subunit were injected individually into sets of oocytes at equal quantities. The same needle was used for the injections of the wild-type and the mutant cRNA to ensure equal quantities of the cRNA injection. The needle was washed several times between injections to avoid cross contamination. The maximal GABA-induced currents were then determined 4 days post-injection (see Supplementary Information-Datasets). To evoke the maximal GABA current in the wild-type and mutant subunits, concentrations of GABA equivalent to 20 to 100 times the corresponding EC_50_ values were used. The average and SEM of the maximal elicited GABA current were first determined for each injection set of the wild-type and mutant subunits. To calculate the relative expression levels of the key mutants, the average of the maximal GABA current in the mutant was divided by the average of the maximal GABA current in the wild-type (Table 4).

### Determination of the maximal current in the co-expressional studies

To evoke the maximal current for the wild-type, mutant, and different wild-type:mutant ratios, concentrations of agonists equivalent to 3 to 100 times the corresponding EC_50_ values were used. To determine the maximal-induced current of the different agonists, each oocyte injected with cRNA of ρ_1_, ρ_I307S/W328I_, ρ_I307S/W328V_, different ratios of ρ_1_: ρ_I307S/W328I_, or that of ρ_1_: ρ_I307S/W328V_ was tested with two applications of GABA, followed by applications of two GABA agonists (I4AA and then ZAPA), anaesthetics, and finally GABA again. Washes of several minutes each were conducted between applications. To determine the relative maxima, the maximal current values for each I4AA, ZAPA, or anaesthetic were then normalized to their respective maximal GABA current values. The current values used in the calculations were limited to those with a magnitude that was less than 1 µA.

### Data fitting and binomial calculations

The data points for the concentration-response relationships were fitted to the following logistic equation:

1 $${\rm{I}}={{\rm{I}}}_{{\rm{\max }}}/(1+{[{{\rm{EC}}}_{50}/{\rm{A}}]}^{{\rm{n}}})$$

where I is the peak current at a given concentration of agonist A, and I_max_ is the maximum current. EC_50_ is the concentration of the agonist yielding a half-maximal current, and n is the slope.

The EC_4_ values were determined based on the concentration-response relationships. The extrapolated values were tested and then adjusted empirically.

The fraction of each sub-population of receptors (containing five, four, three, two, one, or zero mutated subunits) at each ratio was determined using the binomial equation based on the following assumptions: (1) the receptor is a pentamer, (2) the efficiency of the assembly was not affected by the mutations, and (3) the two different stoichiometries present in the receptor chimaeras containing two or three mutated subunits are equivalent in function. The binomial equation is as follows:

2 $${\rm{P}}({\rm{r}})={{\rm{p}}}^{{\rm{r}}}{{\rm{q}}}^{n-r}({\rm{n}}!/{\rm{r}}!({\rm{n}}-{\rm{r}})!)$$

where for a given ratio, r is the number of wild-type subunits incorporated at a given time (e.g., 3); n is the number of subunits in the receptor complex (5); P(r) is the sub-population fraction of the receptor comprising the r wild-type subunits; and p and q are the probabilities of the wild-type and the mutant subunit assimilation, respectively. For example, for the 6:1 ratio of the wild-type to mutant injection, p is equal to 6/7, while q is equal to 1/7.

The percent increases in the GABA currents induced by the anaesthetic (% potentiation) were calculated using the following equation:

3 $$ \% \,{\rm{Potentiation}}=[({{\rm{I}}}_{{\rm{GABA}}+{\rm{Anaesthetic}}}-{{\rm{I}}}_{{\rm{GABA}}})/{{\rm{I}}}_{{\rm{GABA}}}]\ast 100$$

where I_GABA_ is the current value elicited by a given concentration of GABA, and I_GABA+Anaesthetic_ is the evoked current induced by the same concentration of GABA plus the anaesthetic.

### Mathematical simulations

To determine the number of mutated subunits that are required for the activation by the GABA agonist compared to that required for the activation by the anaesthetics, simulations were carried out by assigning experimentally determined values to the sub-population of the homo-oligomers of the wild-type (wild-type-like, close to zero activity) or mutated receptors (mutant-like, close to 100% activity). For the hetero-oligomer receptors containing four, three, two, or one mutated subunits (with unknown activity), depending on the model, either all (homo-oligomeric mutant-like activity) or none weight (wild-type-like activity) was assigned to each receptor sub-population. Three models were considered as follows:

1. The contribution from only the subpopulation of the homo-oligomeric mutant receptors with all weight activity (homo-oligomeric mutant-like activity, ~100%) on the overall current was considered; the remainder of the sub-populations was then speculated to have wild-type-like activity (close to zero).
2. Two receptor sub-populations in the ensemble were simulated to have mutant-like activity. These included the homo-oligomer of the mutated subunit and the hetero-oligomer with four mutated subunits. The remaining four subpopulations were presumed to have wild-type-like activity.
3. Finally, three subpopulations of receptors containing five, four, and three mutated subunits were assumed to exhibit mutant-like activity, while the remaining three subpopulations were instead assumed to have wild-type-like activity (Figs 3 and 4; see Supplementary Information-Datasets for the simulation steps).

To derive the final value of each ratio, the known (homo-oligomers) and the presumed values (hetero-oligomers) of each receptor sub-population were multiplied by the corresponding sub-population fraction present in the ensemble (determined using binomial equation), and the resulting numbers were then summed.

To correct for the differences in the expression levels (determined based on maximal GABA-induced current for mutant relative to that for wild-type, at equivalent cRNA injection), between the wild-type ρ_1_ and ρ_I307S/W328V_ and the ρ_1_ and ρ_I307S/W328Y_ in the simulations, the relative sub-population (fraction) of the receptors containing five, four, three, two, one and zero mutated subunit(s) at each ratio was first estimated using the binomial equation, which assumed the equal assembly of wild-type and mutated subunits. Each subpopulation of receptors was then corrected for the difference in GABA maximal using the following procedure. First, the determined fraction (binomial calculation) of each receptor subpopulation containing 3 or more mutated subunits in each ensemble was multiplied by the relative GABA maximal determined for the mutant (e.g., ~0.5 for ρ_I307S/W328V_, mutant-like expression), while the expression of the receptor subpopulations containing 3, 4 and 5 wild-type subunits was corrected by the wild-type-like expression in terms of GABA maximal (~1). Second, the products from the first step were summed. Finally, each receptor sub-population, corrected for its GABA maximal levels, was normalized to the derived sum in the second step (Supplementary Information-Datasets). Notably, the number of required mutated subunits for the GABA agonist-dependent versus the anaesthetic-dependent activation and the number of mutated subunits needed for potentiation were unaffected if the lower maxima of ρ_I307S/W328V_ or ρ_I307S/W328Y_ were not considered in the calculations of the simulation studies (Supplementary Information-Datasets).

To conduct the simulation of the anaesthetic-dependent potentiation at each ratio, we used experimentally determined potentiation values for the sub-populations of homo-oligomers of ρ_I307S/W328A_ and ρ_I307S/W328Y_ receptors in the ensemble. The values of the potentiation magnitude arising from hetero-oligomeric receptors containing one, two, three, and four mutated subunit(s) (unknown) in the ensemble were estimated by reducing the known potentiation values by ~0.5^n^ (0.47^n^, 0.5^n^, and 0.53^n^ for pentobarbital, 0.57^n^, 0.6^n^, and 0.63^n^ for diazepam), where n represents the number of the wild-type subunits in the pentamer. The numbers (~0.5^n^) used for these simulations were determined using an iterative process. To calculate the final values for the potentiation simulations at each ratio, the known (homo-oligomers) and the presumed (hetero-oligomers) potentiation values for each receptor sub-population were multiplied by the corresponding sub-population fraction present in the ensemble (determined using the binomial equation). The resulting values were then summed. The detailed steps of all simulation procedures corresponding to the I4AA-, ZAPA-, anaesthetic-dependent direct activation, and anaesthetic-dependent potentiation are presented as excel spreadsheets in the Supplementary Information-Datasets.

### Reagents

Drugs and chemical were purchased from Sigma-Aldrich, except for diazepam and propofol (Biomol) and ZAPA (Tocris). Diazepam, propofol, etomidate and midazolam were first dissolved in DMSO. The final solutions of these drugs were prepared by adding the stock to a rapidly agitating solution of OR_2_. Other drugs were directly dissolved in OR_2_.

### Statistics

A student’s t-test (two-tailed, Sigma Plot) was used to determine the statistically significant differences between the values of the anaesthetic-dependent potentiation at different ratios of wild-type to mutant versus the ρ_1_ receptor (Supplementary Information-Datasets). All data are presented as the Mean ± Standard error (s.e.m.).

## Electronic supplementary material

Supplementary Information
